# Association of nail biting and psychiatric disorders in children and their parents in a psychiatrically referred sample of children

**DOI:** 10.1186/1753-2000-2-13

**Published:** 2008-06-02

**Authors:** Ahmad Ghanizadeh

**Affiliations:** 1 Assistant Professor of Child and Adolescent Psychiatry, Shiraz University of Medical Sciences, Hafez Hospital, Shiraz, Iran; 2 Director of Research Center for Psychiatry and Behavioral Sciences, Shiraz University of Medical Sciences, Hafez Hospital, Shiraz, Iran

## Abstract

**Background:**

Nail biting (NB) is a very common unwanted behavior. The majority of children are motivated to stop NB and have already tried to stop it, but are generally unsuccessful in doing so. It is a difficult behavior to modify or treat. The objective of this study was to investigate the prevalence of co-morbid psychiatric disorders in a clinical sample of children with NB who present at a child and adolescent mental healthcare outpatient clinic and the prevalence of psychiatric disorders in their parents.

**Method:**

A consecutive sample of 450 referred children was examined for NB and 63 (14%) were found to have NB. The children and adolescents with nail biting and their parents were interviewed according to DSM-IV diagnostic criteria. They were also asked about lip biting, head banging, skin biting, and hair pulling behaviors.

**Results:**

Nail biting is common amongst children and adolescents referred to a child and adolescent mental health clinic. The most common co-morbid psychiatric disorders in these children were attention deficit hyperactivity disorder (74.6%), oppositional defiant disorder (36%), separation anxiety disorder (20.6%), enuresis (15.6%), tic disorder (12.7%) and obsessive compulsive disorder (11.1%). The rates of major depressive disorder, mental retardation, and pervasive developmental disorder were 6.7%, 9.5%, 3.2%, respectively. There was no association between the age of onset of nail biting and the co-morbid psychiatric disorder. Severity and frequency of NB were not associated with any co-morbid psychiatric disorder. About 56.8% of the mothers and 45.9% of the fathers were suffering from at least one psychiatric disorder. The most common psychiatric disorder found in these parents was major depression.

**Conclusion:**

Nail biting presents in a significant proportion of referrals to a mental healthcare clinic setting. Nail biting should be routinely looked for and asked for in the child and adolescent mental healthcare setting because it is common in a clinical population, easily visible in consultation and relatively unintrusive to ask about. If present, its detection can then be followed by looking for other more subtle stereotypic or self-mutilating behaviors.

## Co-morbidity of psychiatric disorders in children with nail biting and psychiatric characteristic of their parents in a clinical sample

Onychophagia or nail biting (NB) is a behavior with a wide spectrum. It is characterized by putting the nail into the mouth in such a manner that contact occurs between a fingernail and one or more teeth. This could also lead to a damaged or bleeding nails. Sometimes it results in physical damage and is considered as a self-mutilative behavior [2,3]. The gums may even be damaged [4]. Sometimes the nail is bitten until it is lost, the fingers are bitten and the cuticle and the nail-bed skin is chewed [5].

Mild forms of onychophagia had been compared to nervous habits such as fidgeting [6]. Therefore, some studies make a distinction between mild forms and severe forms of nail biting [7]. There is no specific diagnostic category for a number of prevalent habit disorders such as nail-biting in the Diagnostic and Statistical Manual of Mental Disorders, Fourth Edition-Text Revision. Nail biting could be categorized as an 'impulse control disorders not otherwise specified'.

Onychophagia is an unwanted behavior which can make a person nervous in social situations [5]. It is interesting that the majority of children with NB are motivated to stop NB and have already tried to stop it, but have been generally unsuccessful in doing so. The prevalence of nail biting is probably underestimated because of its secrecy and this may lead to under-recognition by medical professionals. The rate of nail-biting in USA preschool children, aged 3 to 6 years, has been as 23% [8]. In an epidemiological study on 4590 school children in India, the rate of NB was reported as 12.7% [9]. A review article reported that up to 33% of children aged 7 to 10 years and 45% of adolescents are nail biters [10]. Another epidemiological study on 5554 children aged 5–13 year old in India showed that girls were more frequently thumb sucking than boys [11]. The rate of NB decreases with the increase in age [8]. In these previous studies, the severity of nail biting was not considered.

A genetic basis for onychophagia has been reported [12]. Onychophagia might be a sign of anxiety and might serve as an anxiety-reducing function [7,13]. Other studies have reported anxiety and nervousness as the etiology of onychophagia [14]. On the one hand one study has reported that it is more than a "nervous habit" and anxious patients more likely perceive their nail biting as a problem [15]. On the other hand, lack of higher anxiety in children with nail biting shows that anxiety is a state rather than a trait [16]. Other researchers have reported that onychophagia is an acquired habit which does not reflect an underlying emotional disturbance [17]. Onychophagia in pediatric dermatology practice may involve an underlying obsessive-compulsive disorder [18]. An older study has reported that there is a higher rate of nail biting in sociopaths as compared to the control population [19]. However, nail biting, especially benign forms of nail biting, can also present without any accompanying psychiatric disorder.

Onychophagia is reported to be a difficult behavior to modify. Long term effects of habit reversal which include awareness training, the practice of an incompatible behavior and relaxation have not yielded impressive results [20]. Furthermore, research has shown that drugs are not effective for treatment of nail biting and habit reversal techniques were not effective in long term [7]. These difficulties may have arisen from insufficient knowledge about NB. Therefore, there is a need to know more about NB in order to reduce or eliminate it. Increasing awareness of co-morbidities that may be associated with NB may ultimately lead to new approaches.

To the best of the author's knowledge, no study has been conducted to investigate psychiatric co-morbidity in children and adolescents with nail biting. This study surveys prevalence of psychiatric disorders and the stereotypic behaviors in a clinical sample of children and adolescents with NB. In addition, it aimed to survey prevalence of the psychiatric disorders in parents of children and adolescents with NB.

## Method

### Sample description

This study was undertaken on a consecutive sample of children and adolescents with nail biting and their parents at the Child and Adolescent Psychiatry Clinic of Shiraz University of Medical Sciences, Fars, Iran. The patients in this consecutive sample were referred to the clinic for different reasons, not just for nail biting. Our average annual patient referral is about 1500. 66 children and adolescents with NB were identified out of a total of about 450 patients referred over 4 months. This represented about one third of the total referrals to the service in this period, a significant proportion of the total referrals. These 66 children were typical referrals in general, with more than two third of them were suffering from disruptive behavior disorders, which matches the proportion of disruptive behavior disorders in our general referrals. Only 3 patients refused to take part in the study because it was very time consuming, leaving a total of 63 children and adolescents who participated in this study together with their parents.

### Measurements

#### Children

Since there are no objective measures to assess nail biting quantitatively, the numbers of days per week whereby the patients would bite their nails was considered as an indicator of severity. Furthermore, duration of NB behavior was elicited with the question, "How many months has he/she bitten his/her nail(s)?" This was assessed based on retrospective self-report of the patient and the estimation of parents. In a pilot study, there was generally good reliability using these methods, and parent and child accounts generally coincided. In the event of a discrepancy between parent and child reports, the parents' report was given priority. In addition, gross physical damage of NB was examined and assessed by the physician.

Psychiatric disorders in children and adolescents were diagnosed by face-to-face interview with them and their parents using Kiddie Schedule for Affective Disorders and Schizophrenia-Present and Lifetime Version (K-SADS-PL) [Farsi version] [21]. K-SADS-PL is a semi-structured diagnostic interview for children and adolescents, based on DSM-IV diagnostic criteria. The K-SADS-PL Farsi version has sufficient validity and reliability for the assessment of child and adolescent psychiatric disorders. It has already been used in many different studies in Iran [22,23]. The stereotypic behaviors including lib biting, bruxism, head banging, skin biting and hair pulling were also surveyed.

#### Parents

The parents were also invited to be interviewed for screening of their own co-morbid current psychiatric disorders using a structural clinical interview by the Schedule for Affective Disorders and Schizophrenia (SADS) and DSM-IV diagnostic criteria [24,25]. The reliability and validity of SADS in Iranian subjects has been previously reported [26,27]. In addition, the parents were asked about lip biting, head banging, skin biting, and hair pulling behaviors. The diagnoses were made by the child and adolescent psychiatrist.

The children and adolescents and their parents were informed about the study objectives and they gave consent to participate in the study. Adequate explanation was given to them: the information collected would be confidential. They would only be used for writing an article, which should improve the life of children with NB and their families through increasing the knowledge about NB. The study was conducted according to the Good Clinical Practice Guidelines, in accordance with the Declaration of Helsinki, 1975, as revised in 2000.

### Analysis

The data were statistically analyzed with SPSS. Chi-squared analysis was used for categorical data and continuous data was analyzed using non-parametric tests. Statistical significance was defined as 5% level.

## Results

63 children and adolescents aged 5 to 18 years old participated in this study. The mean age of the children was 9.4 (SD = 3.3) years. Boys comprised 65.1% of the sample. About 58% of them were the first child in the family and about 31% of them were the only child. The mean duration of NB in the sample was 3.5 (SD = 2.7) years. The duration range was 6 months to 15 years. However, nobody was excluded because of their NB duration.

### Co-morbid psychiatric disorders and the stereotypic behaviors in children and adolescents with NB

Table 1 shows the distribution of co-morbid psychiatric disorders by gender. More boys were suffering from at least one of the psychiatric disorders than girls (X_2 _= 7.9, df = 1, P < 0.01). The most common co-morbid psychiatric disorders in the children were attention deficit hyperactivity disorder (ADHD) 74.6%, oppositional defiant disorder (ODD) 36%, separation anxiety disorder (SAD) 20.6%, enuresis 15.6%, tic disorder 12.7%, and obsessive compulsive disorder (OCD) 11.1%. The rate of major depressive disorder (MDD), mental retardation (MR), and pervasive developmental disorder (PDD) were 6.7%, 9.5%, 3.2%, respectively. There was no case of schizophrenia.

**Table 1:** Association of frequency of the psychiatric disorders by gender in children with nail biting.

Disorder	Boys (n = 41)		Girls (n = 22)	
	
	n	%	n	%
Attention deficit hyperactivity disorder (n = 47)	32	78.0	15	68.2
Oppositional defiant disorder (n = 23)	16	39.0	7	31.8
Conduct disorder (n= 4)	4	9.8	0	0.0
Tic disorder(n- = 8)	7	17.1	1	4.5
Major depressive disorder (n = 4)	2	4.9	2	9.1
Separation anxiety disorder (n = 13)	8	19.5	5	22.7
Mental retardation (n = 6)	5	12.2	1	4.5
Obsessive compulsive disorder (n = 7)	6	14.6	1	4.5
Enuresis(n = 10)	10	24.4	0	0.0
Pervasive developmental disorder (n = 2)	2	4.9	0	0.0
At least one of the above psychiatric disorders (n = 59)	41	100	18	81.8

There was no statistical relationship between the age of the onset of nail biting and the co-morbid psychiatric disorder (Table 2). Also, there was no association between frequencies of nail biting per week with the co-morbid psychiatric disorder. Gross physical damage due to nail biting was not related to the co-morbid psychiatric disorder (Table 3).

**Table 2:** Association of onset age of nail biting by the psychiatric disorders

Disorder	Mean age of onset	Significance*
With ADHD (n = 46)	6.2	U = 267.5, N1 = 46, N2 = 16, p = 0.1
Without ADHD(n = 16)	5.3	
With ODD(n = 23)	5.2	U = 383.5, N1 = 39, N2 = 23, p = 0.3
Without ODD(n = 39)	6.4	
With CD(n = 4)	4.7	U = 96.0, N1 = 58, N2 = 4, p = 0.5
Without CD(n = 58)	6	
With Tic(n = 8)	6.4	U = 188.5, N1 = 8, N2 = 54, p = 0.5
Without Tic(n = 45)	5.9	
With MDD(n = 4)	11.4	U = 188.5, N1 = 4, N2 = 54, p = 0.5
Without MDD(n = 58)	5.6	
With SAD(n = 12)	5.5	U = 53.5, N1 = 12, N2 = 50, p = 0.07
Without SAD(n = 50)	6.0	
With Enuresis(n = 10)	4.9	U = 222.0, N1 = 10, N2 = 52, p = 0.4
Without Enuresis(n = 52)	6.1	
With OCD(n = 7)	6.7	U = 158.0, N1 = 7, N2 = 55, p = 0.4
Without OCD(n = 55)	5.8	
With PDD(n = 2)	7.0	U = 44.0, N1 = 2, N2 = 60, p = 0.5
Without PDD(n = 60)	5.9	
With MR(n = 6)	5.5	U = 124.0, N1 = 6, N2 = 56, p = 0.3
Without MR(n = 56)	6.0	

ADHD = Attention deficit hyperactivity disorder, ODD = Oppositional defiant disorder, CD = Conduct disorder, MDD = Major depressive disorder, SAD = Separation anxiety disorder, MR = Mental retardation, OCD = Obsessive compulsive disorder, PDD = Pervasive developmental disorder

* Mann-Whitney U test.

**Table 3:** Association of gross physical damage of nail by the psychiatric disorder

Disorder		Gross physical damage (%)	Significance
Attention deficit hyperactivity disorder	With (n = 43)	37.2	χ^2 ^= 0.1, df = 1, p = 0.4
	Without (n = 14)	42.9	
Oppositional defiant disorder	With(n = 21)	47.6	χ^2 ^= 1.1, df = 1, p = 0.2
	Without (n = 36)	33.3	
Conduct disorder	With(n = 3)	66.7	-
	Without (n = 54)	37.0	
Tic disorder	With(n = 8)	62.5	χ^2 ^= 0.2, df = 1, p = 0.1
	Without (n = 49)	34.7	
Major depressive disorder	With(n = 3)	0.0	-
	Without (n = 54)	40.7	
Separation anxiety disorder	With(n = 10)	30	χ^2 ^= 0.7, df = 1, p = 0.4
	Without (n = 47)	40.4	
Mental retardation	With(n = 5)	40	-
	Without (n = 52)	38.5	
Obsessive compulsive disorder	With(n = 7)	57.1	χ^2 ^= 0.4, df = 1, p = 0.2
	Without (n = 50)	36.0	
Enuresis	With(n = 8)	50	χ^2 ^= 0.6, df = 1, p = 0.3
	Without (n = 49)	36.7	

*Fisher's exact test

More than half of the children with nail biting (65.1%) had at least one stereotypic behavior. The most common co-morbid stereotypic behavior was lip biting (Table 4). Thirty-seven fathers and 58 mothers were also interviewed. The response rates of the fathers and mothers were 58.7% and 92%, respectively.

**Table 4:** Co-morbidity of the other stereotypic behavior in the children with nail biting

Disorder	Total (%)	Boy (%)	Girl (%)
Lip biting (n = 21)	33.3	26.8	45.5
Bruxism (n = 17)	27	22	36.4
Head banging (n = 11)	17.5	22	9.1
Skin biting (n = 8)	12.7	14.6	9.1
Hair pulling (n = 7)	11.1	7.3	18.2
one or more co-morbid stereotypic behaviors	65.1	58.5	77.3

### Co-morbid psychiatric disorders in parents of children and adolescents with NB

Table 5 shows the frequency of co-morbid psychiatric disorders among the parents of children and adolescents with nail biting. Among the parents who were interviewed, about 56.8% of the mothers and 45.9% of the fathers were suffering from at least one psychiatric disorder. The most common psychiatric disorder concerning the parents was MDD. About 35.1% of the interviewed fathers and 46.6% of the interviewed mothers were suffering from MDD. The rate of anxiety disorders was much lower than the MDD rate. The rate of nail biting was higher than the rate of anxiety disorder regarding the mothers, although it was not statistically significant (X_2 _= 0.04, df = 1, P = 0.8).

**Table 5:** Frequency of psychiatric disorders in parents of children with nail biting

Disorder	Father (N = 37)		Mother (N = 58)	
	
	n	%	n	%
Major depressive disorder	13	35.1	27	46.6
Bipolar mood disorder	1	2.7	2	3.4
Generalized anxiety disorder	0	0.0	3	5.2
Obsessive compulsive disorder	0	0.0	3	5.2
Tic disorder	0	0.0	0	0.0
Nail biting	3	8.1	8	13.8
Post traumatic disorder	1	2.7	0	0.0
Pervasive developmental disorder	1	2.7	0	0.0
Mother with at least one the above psychiatric disorder	-	-	33	56.8
Father with at least one the above psychiatric disorder	17	45.9	-	-

## Discussion

This study of children and adolescents presenting at a mental healthcare clinic showed that 65% of children with nail biting had at least one of the other stereotypic behaviors. More than two-thirds of children who have NB who are referred to a mental health clinic are also suffering from at least one major co-morbid psychiatric disorder. Two-thirds of the interviewed parents were also suffering from at least one major psychiatric disorder, especially MDD. Unfortunately, no study about the co-morbidity of psychiatric disorders in children with NB, co-morbidity of NB in children with psychiatric disorders, or any study about the prevalence of psychiatric disorders concerning parents of children with NB were found to compare with the current results.

The results of this study do not appear to support previous studies which report that onychophagia is a sign of anxiety or that anxiety and nervousness are etiological factors for onychophagia [7,13,14]. Also, these results are not consistent with the study that concluded that onychophagia does not reflect any underlying emotional disturbance [17]. A possible explanation of this lack of consistency is that children and adolescents with psychiatric disorders who also have NB may not be typical of children and adolescents in the community who have NB.

Nail biting is considered by some to be a variant of normal tactile and environmental exploration. However, it should be noted that this behavior causes physical damage and distress as well as a motivation to change, and therefore cannot be considered benign in children. NB is usually associated with psychiatric disorders in this clinical sample. One explanation is that although NB might be associated with anxiety and functions as a tension reduction behavior, this tension and anxiety may be secondary to another psychiatric disorder such as ADHD and its consequences. Affected patients are aware of their habit and admit their continual nail biting, but they seem unable to control it. It is not possible to determine whether the presence of co-morbid psychiatric disorders is a cause or a consequence of NB.

Onychophagia is reported to be a difficult behavior to modify and the treatment results are not as impressive as initially reported [20]. Furthermore, research has shown that drugs are not effective for treatment of nail biting and habit reversal techniques are not effective in the long term [7]. It is possible that low rates of success in treatment might be related to lack of sufficient knowledge about the co-morbidity of psychiatric disorders in children with NB or psychiatric disorders in their parents, and therefore the lack of sufficient resources directed to dealing with underlying causes or maintaining factors. One suggestion would be that future interventional studies on NB should be conducted with special attention to identifying and addressing any psychiatric disorders in these children or adolescents and their parents. This way, it can be determined if treating co-morbid psychiatric disorders in these cases can increase effectiveness of dealing with the NB.

The results of this study suggest that psychiatrists should look for nail biting amongst their patients who present with mental healthcare problems. NB seen in this setting may indicate anxiety. Nail biting, which causes distress to the child and adolescent, may also be an issue that can be used as a way to discuss motivation for change in general.

Care should be taken about generalization of the results because the sample size was relatively low and the participants were exclusively children and adolescents who were referred to the psychiatric clinic for different reasons, not solely for NB. Furthermore, NB duration range was at least 6 months to 15 years. It might show that children with milder forms of NB are less likely to suffer from co-morbid psychiatric disorders and are therefore not as likely to be referred to this clinic; and that this clinical sample consisted of children and adolescents with moderate or severe forms of both nail biting and psychiatric disorders. These might have been key reasons, rather than nail biting per se, for the high co-morbidity rates found in the participants and their parents. Further studies in the general population are recommended.

## Competing interests

The author declares that they have no competing interests.
